# Reply to Miller et al. Comment on “Hermeling et al. Nano-Dry-Melting: A Novel Technology for Manufacturing of Pharmaceutical Amorphous Solid Dispersions. *Pharmaceutics* 2022, *14*, 2145”

**DOI:** 10.3390/pharmaceutics17060715

**Published:** 2025-05-29

**Authors:** Malin Hermeling, Christoph Nueboldt, Roman Heumann, Werner Hoheisel, Joerg Breitkreutz

**Affiliations:** 1 INVITE GmbH, 51368 Leverkusen, Germany; 2 Institute of Pharmaceutics and Biopharmaceutics, Heinrich Heine University Duesseldorf, 40225 Duesseldorf, Germany; 3 Bayer AG, 51368 Leverkusen, Germany

## 1. Letter to the Editor

My fellow authors and I would like to respond to the comment made by Miller et al. regarding our published paper with the title “Nano-Dry-Melting: A Novel Technology for Manufacturing of Pharmaceutical Amorphous Solid Dispersions” [1]. We read the comment and the cited studies with interest and will attempt to correct any misunderstandings and possibly hasty conclusions.

## 2. The Content of Table 1 and KinetiSol^®^

My colleagues and I are sorry to hear that Table 1, as presented, showing the disadvantages and advantages of different technologies, led to this complaint.

We want to point out that the purpose of this table was to show that every method has its advantages and challenges, none of which should be discredited. The table should only be seen as a method of listing and comparison. Nano-Dry-Melting should be seen as an additional method, with benefits but also challenges.

We would like to emphasize that each method listed in Table 1 has its justifications, especially relatively new innovative methods, of which KinetiSol^®^ is an example, that can extend the lists of processable drugs and polymer systems. Which of these methods is used for which active ingredient by a manufacturer depends on many circumstances, which may be technically and economically justified. Table 1 is simply intended to place our new method for preparing amorphous solid dispersions (ASDs) in a comparative context of existing, established methods, without discrediting any of them.

The listed disadvantages of “high mechanical load”, “less stable product”, and “high risk of degradation” were reviewed after accessing the additional studies provided by the authors of the comment.

High mechanical load: We assume that the application of mechanical forces to prepare ASDs in the case of milling, HME, or the Kinetisol^®^ technology often intrinsically involves a high mechanical load on the product. However, we acknowledge that the load is not itself a disadvantage since it need not automatically result in a lower-quality product. Therefore, we concede that the term “disadvantage” should be changed to a rather neutral term, “challenge”. Furthermore, we would like to change the term “lower temperature” in the advantages column to “lower thermal load” and position it at the same height as high mechanical input to emphasize that this advantage is correlated to the “challenge” of the higher mechanical load. This was well described in KinetiSol^®^’s research article [2], which will be cited here.

Less stable product: We came to this conclusion since there are studies that show that compounds from drugs and polymers prepared by milling seem to be fully amorphous when investigated by X-ray diffraction (XRD) or differential scanning calorimetry (DSC) and therefore labeled as ASDs. Even though they are analyzed as “XRD-or DSC-amorphous” they are sometimes not fully homogenized and may still have drug-rich (amorphous) phases or residual crystallites that could act as seeds and induce (re-)crystallization [3]. However, we acknowledge the studies provided by the authors of the comment, which, in part, we failed to consider. In these studies, ASD compounds were prepared with the Kinetisol^®^ technology and analyzed with ssNMR. They clearly show a good homogenization of the drug in the polymer matrix, which convinced us. Thus, we admit that our assessment that Kinetisol^®^ products are less stable was wrong. We would like to withdraw this statement.

High risk of degradation: The thermal treatment of a drug–polymer compound always has a risk of degradation. However, this strongly depends on the temperature and on the time of exposure. For a compound treated with Kinetisol^®^, it is hard to estimate the local temperature of the compound at the moment of high impact and shear. But the time of exposure to this high temperature is certainly short. Therefore, we acknowledge that the risk of degradation should not be classified as high, although it is still present, similarly to with our Nano-Dry-Melting technology. This should be clearly relabeled in Table 1.

We certainly regret the misunderstandings and propose that Table 1 be modified accordingly.

We would like to emphasize again that we do not wish to discredit Kinetisol^®^ Technology in any way but only to introduce Nano-Dry Technology to the scientific and industrial community as another novel method to produce ASDs. These communities should then decide which of the technologies is most advantageous for their specific purposes.

## References

[1] Miller D.A., Kucera S.U., Ellenberger D., Davis D., Williams R.O. (2025). Comment on Hermeling et al. Nano-Dry-Melting: A Novel Technology for Manufacturing of Pharmaceutical Amorphous Solid Dispersions. *Pharmaceutics* 2022, *14*, 2145. Pharmaceutics.

[2] Bhujbal S.V., Mitra B., Jain U., Gong Y., Agrawal A., Karki S., Taylor L.S., Kumar S., Tony Zhou Q. (2021). Pharmceutical amorphous solid dispersion: A review of manufacturing strategies. Acta Pharm. Sin. B.

[3] Thompson S.A., Williams R.O. (2021). Specific mechanical energy—An essential parameter in the processing of amorphous solid dispersions. Adv. Drug Deliv. Rev..

